# Gun theft from private citizens in the US 2020–2024: victims and circumstances from a national survey

**DOI:** 10.1186/s40621-025-00653-2

**Published:** 2026-01-01

**Authors:** David Hemenway, Matthew Miller, Ezra Mason, Samuel Fischer, Deborah Azrael

**Affiliations:** 1 Department of Health Policy and Management, Harvard TH Chan School of Public Health, 677 Huntington Avenue, Boston, MA 02115 USA; 2 Department of Health Sciences, Northeastern University, 360 Huntington Avenue, Boston, MA 02115 USA

**Keywords:** Firearms, Gun theft, Stolen guns, Guns in cars, Gun carrying, Gun storage

## Abstract

**Background:**

This study provides a contemporary estimate of how many adult US gun owners had a gun stolen in the past five years, along with information about the demographic and gun-related characteristics of these victims.

**Methods:**

Data come from the 2024 National Firearms Survey, designed by the authors and conducted by the research firm Ipsos in December 2024. Close to 13,000 (*n* = 12,860) of those invited to take this on-line survey completed it (59% completion rate). Respondents who reported that they personally owned a gun (*n* = 4059) were asked “In the past 5 years, have you had any firearms stolen from you?” Respondents who answered in the affirmative were asked detailed questions about their most recent gun theft incident that occurred in the past five years. Data were weighted to provide national estimates.

**Results:**

Among the 4,059 gun owners in the study, 47 reported having had one or more guns stolen in the past five years, representing a weighted estimate of 1.4% (95% CI 0.9, 1.8) of gun owners, or approximately 1 million adults. Gun owners who had carried a handgun in the month prior to the survey were three times more likely to report having a gun stolen than those who had not carried (OR 3.9, 95% CI 2.1, 7.4). Gun owners who stored a gun in their car (OR 3.6, 95% CI 1.4, 9.2), and those who stored some of their guns loaded and unlocked compared to owners who stored all their guns unloaded and locked (OR 3.6, 95% CI 1.4, 9.6) were also three times more likely to have a gun stolen. Three fifths (60%) of the most recent gun theft incidents reported by respondents occurred in the victim’s home. An additional 15% were from cars, and 25% from all other places. In about a quarter of these thefts (28%), at least one gun had been recovered by the time of the survey. Two-thirds (68%) of respondents reported their most recent gun theft to police (over 80% of non-Hispanic White gun owners compared to about half of other gun owners). One quarter of these theft victims had insurance that covered the firearm.

**Conclusion:**

Gun carrying, keeping a gun in one’s car, and storing guns loaded and unlocked may increase harm to the community by increasing the number of guns that get into the hands of criminals.

## Background

Gun theft is a common method for firearms to get into criminal hands. Estimates indicate that hundreds of thousands of guns in the United States are stolen each year [1–5].

Only a few studies have provided national information about the number and characteristics of gun theft. The data used in these studies come from three main sources: (a) police reports, (b) public surveys and (c) private surveys. All (including the data used in the current study) rely on information provided by the victim—and are subject to the known limitations of self-reported information [6].

Data from reports to law enforcement find that over 95% of all guns stolen in the United States are stolen from private citizens (the other 5% come from licensed gun dealers and interstate shipments) [5]. While more than a hundred thousand private citizens report firearm theft to the police each year, many citizens do not. There is no federal law requiring the reporting of stolen firearms; indeed, no federal agency accepts stolen gun reports from civilians. A minority of states have such reporting laws, but they don’t seem to be strongly enforced—and we could not find studies of how often penalties have been imposed for not reporting.

A large public semi-annual survey, the National Crime Victimization Survey (NCVS), obtains information on thefts both reported and not reported to police. However, neither police reports nor the NCVS provide data about the gun-related characteristics of anyone in the survey (e.g., how many guns they own, whether they carry guns, and how they store their guns).

We believe we wrote the only peer-reviewed journal article providing national information about the gun-related (as well as demographic) characteristics of gun theft victims. That article examined gun theft victimization during 2011–2015 [2]. The current article updates that information for 2020–2024 and provides new national estimates about other aspects of these events (e.g., the percentage of gun thefts from private citizens reported to police, the percentage covered by insurance, and the percentage where the gun was recovered).

## Methods

The 2024 National Firearms Survey (NFS-24), designed by the authors, was conducted in December 2024 by the research firm Ipsos. It is described more fully elsewhere [7]. Briefly, respondents were drawn from Ipsos’s Knowledge Panel (KP), an online panel of approximately 60,000 US adults. The panel, selected on an ongoing basis, is created using an address-based equal probability of selection design to provide samples that are representative of the US adult population. Prior to selection of a study sample, IPSOS adjusts panel base weights to account for discrepancies between panel composition and the distribution of key demographic characteristics of the US population as reflected in the most recent Current Population Survey [8].

For the survey, 21,907 KP members were randomly selected and 12,909 completed the survey (59% survey completion rate). All self-identified gun owners (*n* = 4059) were asked to complete the questions on gun theft and the other questions used in our analyses. Sixteen gun owners did not answer our questions about gun theft, resulting in an analytic sample of 4043. Gun owners were identified through the question “Do you personally own a working gun?” “Gun” was defined as “any firearm, including pistols, revolvers, shotguns and rifles, but not including air guns, bb guns, starter pistols or paintball guns.” Working guns were defined as “guns that are in working order and capable of being fired.” The theft question was “In the past 5 years, have you had any firearms stolen from you?”

For those who answered “Yes,” follow-up questions were then asked about where the most recent theft occurred (my home, a sporting event, a restaurant or bar, a friend or relative’s home, my car, other), whether the most recent theft was reported to police, whether the theft was “covered by insurance,” and whether the firearm was recovered (“since the theft, have you recovered your guns?”). As there is a literature examining police racial bias [9, 10], and to determine whether race might affect the representativeness of police reports, we examined whether there was a difference between the percentage of Non-Hispanic Whites and everyone else reporting a gun theft to the police.

The outcome variable in the analysis was whether the gun owner had 1 + guns stolen in the past 5 years. Six demographic explanatory variables were age (18–45; 45+), gender, ethnicity (White-Non-Hispanic; everyone else), household income (<$75,000; $75,000+), urbanicity (“Which best describes the community that you currently live in?”—rural, suburban, urban), and census region (Northeast, Midwest, South, West). Four gun-related explanatory variables were number of guns owned, carrying guns (“Other than when you may have been hunting or participating in sport shooting, in the past 30 days, have you carried a loaded handgun on your person?”), whether any guns were stored “in my car or other motor vehicle,” and safe gun storage at home (least safe—any gun unlocked & loaded; safest—all guns locked and unloaded; intermediate—everything else).

We conducted all analyses using StataNow 18 (StataCorp), using a weight variable provided by Ipsos. Study-specific poststratification weights provided by Ipsos adjusted for nonresponse, under-or-over coverage from study-specific sampling, and benchmark demographic distributions (from the U.S. Census of Current Population Survey or American Community survey) so that descriptive statistics were representative of the US adult population.

Our research was supported by the Robert Wood Johnson Foundation, and the New Venture Fund. Funders did not play a role in the design, conduct or reporting of the research, or in the decision to submit the manuscript for publication. The Harvard TH Chan School of Public Health and Northeastern University Institutional Review Boards (IRBs)—the human subjects ethics reviews boards– approved the study, determining that studies exclusively using National Firearm Survey data meet the criteria for exemption from IRB review.

The study was performed in accordance with the Declaration of Helsinki. Informed consent was obtained from all participants before enrollment. Participation was completely voluntary, and respondents could skip any questions they preferred not to answer.

## Results

We found that among 4,043 responding adult gun owners in the study, 47 had one or more guns stolen in the past five years, representing a weighted estimate of 1.4% (95% CI 0.9, 1.8) of gun owners (Table 1).

**Table 1:** Correlates of a gun owner having a gun stolen in the past 5 years

	Number of gun owners (unwted)	% had guns stolen (wted) (95% CI)		Unadjusted odds ratios (wted) (95% CI)	
Total	4043		1.4% (0.9,1.8)		
Demographics					
Age					
18–45	1062		2.0% (1.0,3.0)	Ref	
46^	2931		0.9% (0.6,1.4)	0.5* (0.2,0.9)	
Gender					
Male	2721		1.1% (0.7,1.6)	Ref	
Female	1322		1.8% (0.9,2.7)	1.5 (0.8, 3.1)	
Ethnicity					
White	3150		1.0% (0.6,1.4)	Ref	
Hispanic or non-white	893		2.2% (1.1,3.4)	2.2* (1.1, 4.2)	
Income					
<$75,000	2693		2.0% (1.1,2.9)	Ref	
$75,000+	1350		1.1% (0.6,1.6)	0.6 (0.3, 1.2)	
Urbanicity					
Urban	1214		2.0% (1.1,2.9)	Ref	
Suburban	1604		0.8% (0.2,1.3)	0.4* (0.2, 0.9)	
Rural	1214		1.4% (0.5,2.2)	0.7 (0.3, 1.5)	
Region					
Northeast	494		0.4% (0.0,1.0)	Ref	
Midwest	966		1.8% (0.7,2.9)	4.5* (1.0, 32.3)	
South	1745		1.1% (0.5,1.7)	2.8 (0.6, 12.7)	
West	838		1.9% (0.7,3.0)	4.6* (1.0, 21.9)	
Gun-related characteristics					
# Guns owned					
< 6	2935		1.2% (0.8,1.7)	Ref	
6+	959		1.9% (0.8,3.1)	1.6 (0.8, 3.2)	
Carry guns					
No	3019		0.8% (0.4,1.1)	Ref	
Yes	1013		2.9% (1.6,4.2)	3.9* (2.1, 7.4)	
Store gun in car					
No	3624		1.2% (0.7,1.6)	Ref	
Yes	215		4.1% (0.7,7.5)	3.6* (1.4, 9.2)	
Gun storage at home^<^					
Safest	1017		0.6% (0.1,1.2)	Ref	
Intermediate	1637		1.3% (0.6,2.0)	2.0 (0.8, 5.5)	
Least safe	1017		2.3% (1.2,3.4)	3.6* (1.4, 9.6)	

^*^Significance based on 95% CI

^^^Age range 46–98

^<^Safest: all guns unloaded and locked

Least safe: 1 + gun loaded and unlocked

Intermediate: all other situations

Between 2020 and 2024, on average, there were some 260 million adults, of whom approximately 28% were gun owners. Among these gun owners, the 1.4% represents about 1 million who were victims of gun theft in the past five years.

Demographically, certain types of gun owners were more than two times as likely to have a gun stolen compared to other gun owning groups: younger (aged 18–44 compared to 45+); Hispanic or non-white compared to white; urban compared to suburban; and other regions compared to New England (Table 1).

Among gun-related characteristics, the types of gun owners more than two times as likely to have a gun stolen were those who had carried a handgun in the 30 days prior to the survey (OR 3.9, 95% CI 2.1, 7.4), who stored a gun in their car (OR 3.6, 95% CI 1.4, 7.4); and those whose gun storage included at least one gun that was loaded and unlocked(compared to those whose guns were all loaded up and unloaded) (OR 3.6, 95% CI 1.4, 9.6) (Table 1).

Well over half (60%) of the most recent gun thefts reported by respondents occurred at home, 15% were from cars, with 25% from all other places (e.g., restaurant, bar, sporting event, home of friend or relative) (Table 2). In about a third of the incidents (31%) the firearm loss was covered by insurance, and in 28% of the incidents at least one gun had been recovered by the time of the survey. Over two-thirds of the gun thefts (68%) were reported to the police (Table 2).

**Table 2:** Circumstances of gun thefts (2020–2024)

Location of gun thefts	Weighted %
Home (excluding vehicle)	60%
Own Car	15%
Home of friend or relative	8%
Other	18%
Theft reported to police	68%
Theft covered by insurance	31%
Stolen firearms recovered	28%

Comparing the likelihood of reporting a gun theft to the police between non-Hispanic white and other gun owners, we find 81% of non-Hispanic white respondents said they reported their gun theft to police, while 53% of other gun owners did (not shown).

## Discussion

The United States has much higher rates of criminal gun use than other high-income countries—higher rates of gun robbery, intentional woundings and killings (homicides). A federal law requires that the initial purchase of firearms occur through a licensed dealer and requires a federal background check. Unfortunately, many guns are obtained by individuals who could not pass a background check; a common method is via gun theft [11]. Stolen guns are more likely than other guns to be recovered in crime [4].

Our finding that approximately 200,000 gun owners per year have had a gun stolen from them is roughly consistent with estimates from both police reports and the NCVS. Data from the Federal Bureau of Investigation National Crime Information Center (NCIC) show that between 2019 and 2023 [5], an annual average of 168,000 private citizens reported a gun theft to police. The NCVS survey estimated that from 2018 to 2022, somewhat under 150,000 non-fatal victimizations per year involved the theft of a firearm [3]. Since the turn of the 21st century, the NCVS yearly estimates of the number of yearly gun theft incidents fluctuated between approximately 100,000 and 200,000 [3].

We found that the demographic and gun-related characteristics of gun owners most likely to report having a gun stolen on our 2015 and 2024 surveys were quite similar [2]. For example, in both five-year periods, these demographic groups were more than twice as likely to have their guns stolen: younger (aged 18–45) compared to older adults (aged 46+); Hispanic and non-white compared to non-Hispanic white respondents; urban compared to suburban residents; and South and West residents compared to those living in the Northeast.

In addition, in both periods those gun owners who carried a gun in the past month (compared to those who did not carry a gun), stored a gun in their vehicle and kept at least one loaded and unlocked firearm in their home were more than twice as likely to have a gun stolen. Studies evaluating carrying gun laws provide support for our findings connecting carrying guns and gun theft; recent studies find increasingly permissive gun carrying laws are associated with increased gun theft incidents (reported to police) [12, 13]. Or put another way, our results support the notion that strengthening gun carrying laws might help reduce gun theft. The association between gun carrying (as well as keeping a gun in the car, and less safe gun storage) and gun theft makes sense as studies of criminals indicate that gun theft generally appears to be more a function of opportunity than a concerted effort to obtain weapons [14].

Gun storage has been receiving increasing study by scholars. Consistent with work done by us and others [15, 16], we find relatively low rates of secure (unloaded and locked) gun storage among gun owners in our survey. Storing firearms unlocked and loaded has been associated with both suicide [17, 18] and gun accidents [17] among youth. Our studies indicate an additional public health problem—the increased likelihood of guns getting into criminal hands. A recent study finds that, perhaps not surprisingly, “widespread, bipartisan aversion exists to neighbors…storing guns insecurely” [19].

Our finding that most (60%) gun thefts occurred from someone’s home is broadly consistent with available police data. For the five years from 2018 to 2022, data from the FBI’s National Incident-Based Reporting System (NIBRS) showed that about 2/3 of gun thefts were from residences [4]. The NIBRS data for those years come from police departments covering about ¼ of the US population. The NIBRS data also showed a large increase in gun thefts from cars in recent years in urban areas [4, 20]. Our sample size was too small to draw any conclusions about changes in gun theft from cars in the past 5 years.

We found that about 2/3 of people whose guns were stolen reported it to the police. The most recent readily available NCVS results show that 75% of gun thefts were reported to a law enforcement agency [21]. Our study found that in 28% of gun theft incidents, one or more of the stolen guns had been recovered. This is consistent with police reports that between 2019 and 2023, 30% of the guns stolen had been recovered by police [21].

The percentage of gun thefts covered by insurance (31% in our study) does not seem to have been reported before. A recent paper claims that insurance companies “may be in a unique position to mitigate firearm-related deaths” [22]. However, little is known about the financial effects on insurance companies of firearm-related harms, and we could find no empirical studies on insurance payoffs for firearm theft, or on incentives insurance companies may be providing for safe firearm storage.

Our study has various limitations. One is that, although we questioned over four thousand gun owners, the number that reported to us that a gun was stolen (*n* = 47) was too small for many disaggregated analyses. For example, comparing those owners who reported the theft to the police versus those who did not, was comparing 31 people to 16 people. The biggest difference across demographics was that Hispanic and non-white gun owners appeared to be less likely than non-Hispanic white owners to tell the police when their guns were stolen– suggesting that NIBRS data on the relative likelihood of a gun theft by race of victim might be misleading—but that difference was not statistically significant (not shown).

Another limitation is that the data are cross-sectional, so while the results can be consistent with various theories, they do not necessarily indicate causation. The results do not show, for example, that guns were stolen because they were loaded and unlocked, or even that they were stolen while they were loaded and unlocked. All that is shown is that owners who currently store some of their guns loaded and unlocked are the ones most likely to report on surveys that they were the victim of a gun theft.

We also only ask *current* gun owners about gun theft. We thus miss someone who owned guns during the previous 5 years, was a victim of gun theft, but presently does not own a gun. We also do not ask individuals about gun theft who are not gun owners but live in a household with a gun owner. Our interest is in the number of gun owners who had guns stolen, not in the number of people who experienced a gun theft in their household.

Perhaps the biggest limitation of the study is that–like all national data on gun theft from private citizens (e.g., police reports, NCVS)—it relies on self-reports, and self-reports may not be completely accurate. A particular problem with self-report for this study is, given that being a victim of gun theft is a very rare event, there is a danger that inaccurate responses to our Yes/No screener question of whether any guns were stolen might lead to more false positives than false negatives [23]. Fortunately, a positive response to having a gun stolen seems less likely to be subject to positive presentation bias than, say, effectively using a gun in self-defense to thwart a criminal, or financially supporting the National Rifle Association or gun control organizations. Further, our estimates of the number of gun thefts reported to police are consistent with the number of gun theft incidents reported by police agencies [5].

Given how few survey respondents report gun theft, and thus the undue leverage even a single outlier could have on the number of guns stolen or the number of times guns were stolen, we restrict our estimates to the number of gun owners who had guns stolen guns in the past five years. Assuming complete accuracy in the self-reports, our estimate of the number of gun *owners* having had guns stolen from them in the past 5 years can be considered a lower bound for the total number of *guns* stolen (i.e., more than one gun can be stolen in any incident and some gun owners can have more than one gun theft incident over a 5-year period).

There is still much to learn about the circumstances and effects of gun theft. For example, much more needs to be known about who is stealing guns, and the underground markets through which stolen guns often make their way into the hands of the perpetrators who use them in assaults and robberies [4]. We need to know more about many possible policy levers, such as new technology that can make guns less easy to steal from cars, and how to engage licit gun owners to become more part of the solution rather than part of the gun theft problem [24].

## Conclusions

Our main findings are that approximately 1 million U.S. gun owners had one or more guns stolen in the past five years, that most of these people did not have insurance for their stolen guns, and most did not recover their guns. It also seems clear that, as was indicated in our previous study [2], certain types of gun owners are at higher risk for becoming victims of gun theft– younger age, Hispanic/non-white, not living in the suburbs, not living in the Northeast, carrying guns, storing guns in cars, and storing them loaded and unlocked.

In other words, not only does less safe gun storage increase the likelihood of gun accidents and gun suicide of children and youth [17, 18], but it may increase the harm to others in the community by allowing guns to get into the hands of people who use them in crime. Gun carrying may both increase the risk of violent crime [25] and the likelihood of guns being stolen. And, of course, your gun cannot be stolen from your car if you do not keep it there.

## Data Availability

The data analyzed during the current study are available from the corresponding author on reasonable request.

## Abbreviations

ATF: Bureau of Alcohol, Tobacco, Firearms and Explosives
IRB: Institutional Review Board
KP: Knowledge Panel
NCIC: National Crime Information Center
NCVS: National Crime Victimization Survey
NFCTA: National Firearms Commerce and Trafficking Assessment
NFS: National Firearm Survey
NIBRS: National Incident-Based Reporting System
